# Novel mtDNA methylation-associated prognostic signatures in colorectal cancer

**DOI:** 10.3389/fonc.2025.1684770

**Published:** 2026-01-02

**Authors:** Qiang Wang, Zhongsheng Chen, Lianghe Li, Jiandong Zhang, Qing Li, Wei Zhan

**Affiliations:** Department of Colorectal Surgery, The Affiliated Hospital of Guizhou Medical University, Guiyang, Guizhou, China

**Keywords:** colorectal cancer, drug sensitivity, immune infiltration, mitochondrial DNA methylation, prognostic genes

## Abstract

**Background:**

Given the high morbidity and mortality of colorectal cancer (CRC), as well as its challenging treatment, the present study was conducted to identify reliable prognostic signatures for predicting clinical outcomes.

**Methods:**

This study identified key genes through differential expression analysis and cluster analysis on CRC transcriptomic data from public databases and mitochondrial DNA methylation-related genes (MDM-RGs) from previous literature. Cox analysis and machine learning were used to define prognostic genes and build a prognostic model, followed by the establishment of a nomogram to assess the risk score as a prognostic indicator. Furthermore, the impact of prognostic genes was explored by employing immune infiltration, antitumor immunoassay, medication sensitization assay, and prognostic gene dependency analysis. In addition, the expression of MDM-RGs in CRC was assessed by qPCR and Western blotting.

**Results:**

Consequently, this study identified three prognostic MDM-RGs (TINAG, EPHB2, and FCN3), and a risk model was constructed based on these three prognostic genes. A nomogram was created to predict CRC prognosis clinically. Significant differences in risk scores were observed across subgroups, particularly in stage and T stage groups. This study observed disparities in immune cells involving lymphocytes and memory cells, with the identification of 92 pharmaceuticals showing intergroup significant differences. Sorafenib, Salubrinal, and Roscovitine were positively correlated with the risk score, whereas WO2009093972 was negatively correlated. Additionally, this study identified several target genes such as FBXO25 with TINAG, CCDC28A with EPHB2, and SH2D6 with FCN3, with subsequent validation achieved through qPCR and western blotting.

**Conclusions:**

In conclusion, this study identifies three prognostic genes, providing new insights into CRC pathogenesis and potential therapeutic strategies.

## Background

Colorectal cancer (CRC) is one of the cancers with the highest incidence and mortality among all cancers that seriously threatens human life and health. The incidence is rising rapidly among young people (1). It is generally managed by surgery and drug therapy, especially chemotherapy, imposing a serious economic burden on patients (2). Meanwhile, drug therapy is inefficient, toxic, and dependent on specific gene mutation status, with relatively low overall benefit for stage II/III CRC patients after radical surgery (3). Currently, the survival of treated CRC patients is still not optimistic in the context of a lack of effective and specific biomarker for the prognosis of CRC (4). Therefore, it highlights the necessity for early screening and identification of more accurate prognostic indicators for CRC or its precancerous lesions (5). There is an urgent need to establish reliable prognostic models that can predict clinical outcomes. Existing studies have confirmed that the diagnosis and treatment of tumors may be benefited from the development of new diagnostic and therapeutic methods related to genes (6, 7). Prognostic genes can be useful for predicting disease progression, survival, and therapeutic response, distinguishing high-risk and low-risk populations, assisting in personalized medicine, optimizing treatment strategies, and improving patients’ clinical outcomes and quality of life. For instance, Xiao et al. discovered and validated a novel combined prognostic feature associated with immunity and metabolism. This feature can classify colorectal cancer (CRC) patients into low-risk and high-risk groups, thereby preventing adverse survival outcomes (8). Additionally, they found that patients with high expression of the immune-related gene SPP1 tend to have a worse prognosis (9). At this stage, further research is needed to investigate its clinical significance, despite the existence of data on the prognostic genes of CRC. Therefore, it is still important and urgent to search for effective prognostic genes for CRC patients and explore their clinical significance.

DNA methylation and demethylation, as one of the most important epigenetic modifications, have been highly valued for their roles in a biotic stress response. Changes in mitochondrial DNA methylation and hydroxymethylation have been observed in breast cancer, cardiovascular disease, diabetes, neurodegenerative disease, and a series of other diseases (10). Cancer stem cell-like cells are highly methylated and maintain low mtDNA copy numbers, leading them to rely on aerobic glycolysis to promote cell proliferation (11). A low mitochondrial membrane potential may induce mitochondrial DNA methylation via DNA methylation transcription factors, thereby promoting epithelial–mesenchymal transition and, consequently, the occurrence and metastasis of cancer (12). It has been reported that the mitochondrial genome is involved in maintaining the malignant behaviors of cancer cells, with the detection of alterations in mitochondrial DNA in CRC (13, 14), leading to increased cell proliferation, decreased apoptosis, relative cell cycle arrest in the G0/G1 phase, etc. Therefore, in-depth research on mitochondrial DNA methylation is beneficial for understanding CRC pathogenesis, thus providing insights into the prognosis assessment and treatment of CRC.

With respect to the above, this study was conducted on the basis of transcriptomic data and clinical data sourced from TCGA-CRC, a public database, combined with the use of machine learning algorithms to screen mitochondrial DNA methylation-related genes (MDM-RGs) associated with CRC prognosis. This study continued to conduct in-depth analysis of the interactions between these prognostic genes and the immune microenvironment, with purposes of elucidating the correlation between prognostic models and clinical pathological features, as well as exploring the functions and regulatory mechanisms of these genes in different subgroups. Finally, this study evaluated the association between prognostic genes and chemotherapy, with the identification of the target with the strongest correlation between prognostic genes through gene dependency analysis. In addition, real-time quantitative PCR (RT-qPCR) and Western blotting were utilized on clinical samples to investigate the expression level of MDM-RGs in CRC and corresponding prognostic potential. Altogether, it is anticipated that these results may provide a solid foundation for deeper understanding of the molecular mechanisms of CRC and the development of novel therapeutic strategies, thereby facilitating innovation in CRC treatment and realizing personalized treatment.

## Methods

### Data acquisition

The University of California, Santa Cruz (UCSC) Xena database (https://xena.ucsc.edu/) was accessed to acquire the gene expression profiles, clinical data, and survival outcomes of the TCGA-CRC cohort (15). This study further merged the TCGA-CRC dataset, which included 539 colon cancer (COAD) tissue samples and 178 rectal cancer (READ) tissue samples, resulting in 632 samples (tumor:normal = 584:48). The dataset GSE87211, where sequencing was conducted on the GPL13497 platform, comprising tumor tissue samples from 203 READ patients (normal tissue samples were not used), was available from the Gene Expression Omnibus (GEO) database (https://www.ncbi.nlm.nih.gov/geo/), a database covering multiple fields including tumor and non-tumor areas (15). The dataset was designed with distinct purposes to optimize the model performance and ensure generalization capability. For the tumor samples in the TCGA-CRC dataset, it was partitioned into a training set and a testing set (7:3) with 409 samples and 175 samples, respectively, whereas the GSE87211 dataset was utilized as the validation set. In addition, eight MDM-RGs (DNMT1, DNMT3A, DNMT3B, SLC25A26, METTL4, NRF1, PPARGC1A, and PRKAA1) were obtained from the literature (**Supplementary Table 1**) (16–21). In this study, MDM-RGs referred to genes for which there is evidence of their ability to directly regulate or indirectly affect mtDNA methylation.

### Weighted gene co-expression network analysis

In this study, key module genes were uncovered by weighted gene co-expression network analysis (WGCNA). Firstly, the scores for MDM-RGs in the TCGA-CRC dataset were worked out via the single-sample gene set enrichment analysis (ssGSEA) algorithm using the R package “GSVA” (v1.46.2) (22). The next step was the analysis of differences in MDM-RG scores between tumor and normal samples. Subsequently, tumor samples from the TCGA-CRC dataset were segregated into high-risk and low-risk groups based on the optimal thresholds derived from MDM-RG scores. The survival disparities between groups were assessed using a log-rank test, with the screening of genes with significant differences for further analyses. After that, by employing the “WGCNA” package (v 1.71), WGCNA profiling was performed on tumor samples from this dataset (23), with MDM-RG scores as the trait. Furthermore, to detect and eliminate outliers, this study utilized a clustering analysis for all samples hierarchically based on the Euclidean distance of their expression profiles. Subsequently, the optimal soft threshold was set based on the scale-free fitting index (signed R²) and mean connectivity (close to 0). Gene adjacency was computed to determine gene similarity and hence derive a gene dissimilarity coefficient for constructing a hierarchical clustering tree of genes. Then, connections between traits and modules were established by using the MDM-RG scores associated with traits. In addition, correlation coefficient matrices were computed between module eigenvectors and traits, with the determination of the key module by identifying the best relevance on the basis of the criteria of |cor| > 0.3 and *p* < 0.05.

### Identification and functional annotation of differentially expressed genes

DEGs between CRC and normal samples within the TCGA-CRC dataset, foundational to the analysis, were identified using the “DESeq2” R package (v 1.42.0) (24), with the use of the criteria of adj. *p* < 0.05 and |log2 fold change| ≥ 1. To visualize the results, a volcano plot, with the “ggplot2” package (v 3.4.4) (25), was generated and a heatmap created using the “ComplexHeatmap” package (v 2.14.0) (26). Subsequently, by intersecting DEGs with key module genes (https://cran.r-project.org/web/packages/ggvenn/index.html), the “ggVenn” package (v 0.1.10) was exploited for candidate genes. Then, using the “clusterProfiler” R package (v 4.7.1.003) (27) (*p.adj* < 0.05), the Kyoto Encyclopedia of Genes and Genomes (KEGG) (28–30) and Gene Ontology (GO) analyses were conducted on the screened candidate genes to explore the functions of these candidate genes.

### Identification of prognostic genes

Univariate Cox analysis was conducted on candidate genes utilizing the “survival” R package. (v 3.5-7) (https://cran.r-project.org/package=survival) (HR≠1 & *p* < 0.05) to pin down the effect of candidate genes on the survival status in the training set. Subsequently, the identified genes were subjected to the proportional hazards (PH) test to exclude genes with *p* < 0.05. Then, the least absolute shrinkage and selection operator (LASSO) analysis was conducted on genes obtained above using the “glmnet” package (v 4.1-8) (31) to further downsize the number of genes scientifically. Ultimately, genes derived from LASSO regression were involved for multivariate Cox analysis to appraisal of their impact on CRC survival. Genes meeting the criteria of HR≠1 and *p* < 0.05 would be selected as prognostic genes.

### Evaluation and validation of prognostic models

In the training set, based on the expression levels of prognostic genes and the hazard ratios, risk scores for CRC patients were calculated to give an insight on the availability of prognostic genes. The risk model was constructed based on the calculation of the risk score using the following equation:

(1) $$riskscore=\sum_{i=1}^{n}coef(gene_{i})\times expr(gene|\;|i)$$

where “Coef” denotes the each gene’s risk coefficient, and “expr” represents the gene expression level. Patients from the training set were categorized into high-risk and low-risk groups using the optimal threshold. The Kaplan–Meier survival curves were plotted to estimate the capacity of the risk model by employing the “survminer” R package (v 0.4.9) (33). Furthermore, based on the prognostic model derived from prior analysis, the receiver operating characteristic (ROC) curve was drawn to appraise the validity of the prognostic model by the “timeROC” package (v 0.4) (32), with nodes at survival times of 1, 2, and 3 years. An area under the ROC curve (AUC) of >0.6 indicated moderate performance of the prognostic model. Similarly, risk scores were computed for CRC patients and ROC curves were plotted in the testing and validation cohorts. Finally, patients were further divided into the same two groups as before using optimal thresholds, followed by the analysis of intergroup survival differences.

### Standalone prognostic analysis and nomogram building

The efficacy of the risk model as an autonomous prognostic factor was conducted by incorporating the risk score, age, gender, and pathologic TMN in the univariate Cox analysis (*p* < 0.05) with a proportional hazards (PH) assumption test (PH > 0.05). Then, using the “rms” package (v 6.7-1) (https://cran.r-project.org/web/packages/rms/index.html), the nomogram was generated to predict the survival of CRC patients in TCGA-CRC. Finally, the calibration curve was plotted to further measure the predictive capacity of the nomogram.

### Characterization of clinical profiles

To further explore the distribution of clinical features within different risk groups, the proportions of allocation of a wide range of clinical factors (age, gender, stage, and pathologic TMN) among patients in both groups were statistically analyzed in the training set. Subsequently, to clarify the intrinsic mechanism of risk scores relating to the clinical features of CRC patients, variations in risk scores across diverse sets of clinical characteristics were measured among subgroups of each clinical feature.

### Functional annotation for gene sets

With the “c2.cp.kegg.v7.0.symbols.gmt” as the reference gene set (https://www.gsea-msigdb.org/gsea/msigdb), the biological functional variances between patients in different groups were elucidated by the MSigDB database. The R package “DESeq2” was used for intergroup comparison to get the variance of CRC samples from the training set. The outcomes were then arranged in descending order according to the value of log2FC. Additionally, to comprehend the disparity in biological function between groups, gene set enrichment analysis (GSEA) on both groups within the training set samples was carried out using the “clusterProfiler” R package (|normalized enrichment score (NES)| > 1 and *p* < 0.05).

### Clarification of the immune microenvironment

Initially, to identify the impact of the tumor immune microenvironment on disease progression, this study evaluated the levels of immune cell infusion within both groups in the training set using three immune infiltration algorithms (EPIC, QUANTISEQ, and MCPCOUNTER) within the “IOBR” R package (v 0.99.9) (34). To clarify disparities in immune cell infiltration across the high-risk and low-risk groups, differences in immune cell composition between groups were assessed by employing three distinct algorithms for immune infiltration analysis. Lastly, using the R package “psych” (v 2.4.1) (https://cran.r-project.org/web/packages/psych/index.html), Spearman correlation analysis was carried out in the TCGA-CRC dataset to examine the relationship between risk scores and immune cell populations identified earlier. The minimum thresholds were set at |cor| > 0.3 and *p* < 0.05.

### Antitumor immunoassay

In general, the cancer-immunity cycle refers to a conceptual framework delineating a sequence of progressive steps, which involve the release of antigens from cancer cells, subsequent presentation of these antigens, priming and activation of immune responses, migration of immune cells to the site of tumor, infiltration of immune components into tumor tissues, recognition of cancer cells by T cells, and the elimination of cancerous cells ultimately. To evaluate the anticancer immune reaction in patients from different groups, we got access to the tumor immunophenotype (TIP) (http://biocc.hrbmu.edu.cn/TIP/) to source the genes regulating each step. Furthermore, to develop an adequate comprehension of the mechanisms of cancer immunity, the enrichment scores of the screened regulatory genes at each step were computed using the ssGSEA algorithm via the “GSVA” package (v 1.46.2) (22). The final step was an additional evaluation of the discrepancies in gene enrichment scores between groups (*p* < 0.05).

### Tumor mutation analysis

To gain insights into the somatic mutations between the high-risk and low-risk groups, the CRC mutation data were downloaded from the training set. Somatic mutations in all samples (n=409) from the training set were analyzed using the maftool package (v 2.22.0). Moreover, waterfall plots of the top 20 most frequently mutated genes in the high-risk and low-risk groups were generated using the oncoplot function with default parameters (35). Subsequently, waterfall plots were generated to visualize the top 20 most frequently mutated genes in the two risk groups.

### Pharmaceutical sensory analysis

In order to further evaluate the association between prognostic genes and response to chemotherapeutic drugs, the IC50 values of 138 common chemotherapeutic and molecular targeted drugs for all patients in the training set were calculated using the “pRRophetic” package (v 0.5) (36). The “pRRophetic” package referred to training data from databases such as GDSC/CTRP/CCLE during calculation (tissueType = “allSolidTumors”) and maintained the default gene expression normalization and batch effect processing during the prediction process (with log transformation and mean processing of duplicate genes employed internally in pRRophetic). Differences in IC_50_ expression of common chemotherapeutic drugs between the high-risk and low-risk groups were compared using the Wilcoxon rank-sum test (*p.adj* < 0.05, Benjamini–Hochberg (BH) correction). Subsequently, the correlation between the risk score and the IC_50_ value of the drugs were calculated with significant differences (adjusted *p* < 0.05, BH correction, |cor| > 0.3).

### Assessment of prognostic genes

To further evaluate potential target genes, the DepMap CRISPR database (https://depmap.org/portal/) was used to identify genes with gene essentiality in CRC cell lines. The correlation between the prognostic genes and genes with gene essentiality was assessed via Spearman correlation analysis. To screen for meaningful potential target genes, a threshold of |cor| > 0.3 was set. Within this framework, “potential target genes” were defined as genes that are highly dependent (genetic dependency/lethality) in CRC cells and correlated with prognostic genes. In addition, to give an insight into the expression patterns of these genes during disease progression, the mRNA levels of prognostic genes between CRC and control samples were analyzed in the TCGA-CRC dataset.

### Validation of the expression levels of prognostic genes at the clinical level

The present study continued to conduct a series of relevant experiments to further validate the expression of prognostic MDM-RG in CRC at the mRNA and protein levels. This experiment was initiated by collecting tumor specimens and their corresponding normal colorectal tissues from 18 clinical samples of CRC after surgical resection in the Affiliated Cancer Hospital of Guizhou Medical University during July 26 to August 30, 2024. The harvested tissues were used for extracting mRNA and protein. All patients did not receive neoadjuvant chemoradiotherapy or antitumor drug treatment before surgery, with the confirmation of adenocarcinoma in all cases through preoperative pathological examination. This study was performed according to the Declaration of Helsinki and approved by the Hospital Ethics Committee (Approval No. FZ 2024-05-207). Prior to collecting tissue samples, informed consent forms were obtained from each patient.

### RT-qPCR

Changes in mRNA transcription levels of the target genes in the collected samples were detected using the SYBR Green I Real Time PCR method. Total RNA was extracted from CRC tissue and its corresponding normal colorectal tissue using TRIzol reagent (Invitrogen). Following reverse transcription of RNA into cDNA using a reverse transcription kit (Kangwei reagent (Cat # CW2582M)), RT-qPCR was performed using a RT-qPCR kit (Cat # MQ00401S, Mona Biotech). Using a fluorescence quantitative PCR instrument (Bio-Rad, the United States), differences in prognostic gene expression levels between tumor and control samples were calculated using the ^2−△△CT^ method, with three technical replicates. The specific primer sequences are shown in **Table 1**, with GAPDH serving as an internal reference.

**Table 1:** Primer sequences for target genes.

Genes	Sequences
GAPDH	TCAGTTCGGAGCCCACACGC
	ACCAGGGAGGGCTGCAGTCC
EPHB2	CTCGAGGGCAGACAGGAGGATAGTTGTT
	TGCGGCCGCGGGCGCTGATGTAGTTC
TINAG	GTTCCAAGGAGAAGCCCACA
	CCGGTCCACATTCTCTGGAA
FCN3	CCCAGTCTTTTGTGACATGGA
	CCTGCTCTGTAGGAGGACCA

### Western blot

Total proteins were extracted from CRC tissues and their corresponding adjacent tissues using protease inhibitor-containing RIPA buffer. Then, the extracted protein was quantified using the BCA protein detection kit (Biotech, C500053-0050). Then, an equal amount of sample was taken for separation by 12% SDS-PAGE electrophoresis. After transfer onto a PVDF membrane, the next step was soaking in Tris-buffered saline containing Tween 20 (TBST) at room temperature for 2 h. Subsequently, the processed PVDF membrane was incubated overnight with specific primary antibodies at 4 °C. After washing in TBST for 15 min, the PVDF membrane was incubated again with peroxidase (HRP)-labeled secondary antibody at room temperature for 2 h to detect the immune response bands. β-Actin was an internal reference. Specifically, the EPHB2 antibody (W83277-1-RR), β-actin antibody (81115-1-RR), and HRP-labeled rabbit secondary antibody (RGAROO1) were all purchased from Wuhan Sanying Biotechnology Co., Ltd. (Wuhan, Hubei, China).

### Statistical analysis

Using the R programming language (v 4.2.2), variations between groups were assessed by bioinformatics analyses involving the Wilcoxon rank-sum test. Fisher’s exact test was utilized to determine the statistical significance with a threshold of *p* < 0.05. Data were processed using GraphPad Prism 9.0 and SPSS 27.0, and a two-sided t-test was used to evaluate intergroup differences. The difference with *p* < 0.05 was determined to be statistically significant.

## Results

### Identification of candidate genes in CRC

**Figure 1:**
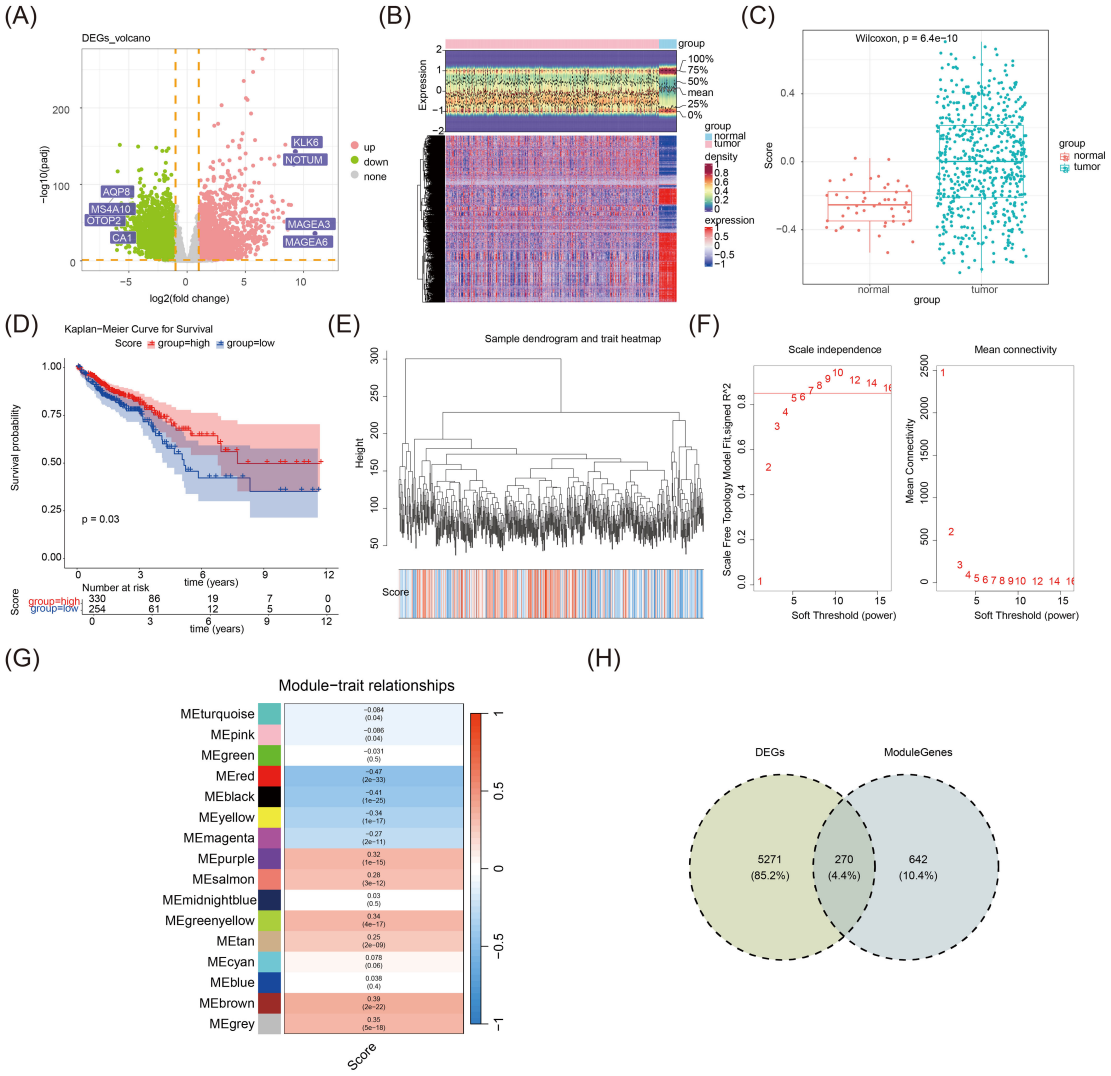
**(A)** Volcano map of TCGA-CRC dataset differences. In the figure, each dot represents one gene. Pink dots indicate genes that are upregulated in expression, whereas green dots indicate genes that are downregulated in expression. The x-axis denotes log2FC, which is the logarithm of the fold change in gene expression levels. The y-axis denotes -log10 (padj), which is the negative logarithm of the adjusted *p*-value. **(B)** Differential heatmap of the TCGA-CRC dataset. Red indicates upregulated gene expression, whereas blue indicates downregulated gene expression. The darker the color, the higher or lower the expression. **(C)** Box plot based on MDM-RG-related gene scoring between tumor and normal samples. **(D)** KM survival curves of MDM-RG high-risk and low-risk groups in the TCGA-CRC dataset. **(E)** Sample level clustering. **(F)** Soft threshold filtering. **(G)** Correlation heatmap between modules and traits. Red indicates a positive correlation, and blue indicates a negative correlation. The darker the color, the stronger the correlation. **(H)** Venn diagrams of the intersection between key module genes (912) and DEGs (5,541).

The analysis of DEGs in the TCGA-CRC dataset identified 5,541 DEGs, with 2,684 upregulated and 2,857 downregulated genes (**Figures 1A, B**). The MDM-RG scores were significantly higher in the tumor group than in the normal group (**Figure 1C**). Then, tumor samples were stratified into high-risk and low-risk groups based on the optimal thresholds derived from MDM-RG scores, revealing a statistically significant distinction between groups (*p* = 0.03), with a higher survival rate found in the high-scoring group (**Figure 1D**). Subsequently, through WGCNA, outlier samples were removed by hierarchical cluster analysis, with no significant outlier samples (**Figure 1E**). The soft threshold β, crucial for determining gene correlations, was found to be most appropriate at 7 (**Figure 1F**). Then, 15 co-expression modules were identified, with the key module (MEred) containing 912 genes selected based on its highest relevance (|cor| > 0.3 & *p* < 0.05) (**Figure 1G**, **Supplementary Table 2**). Subsequently, to identify genes that are not only associated with MDM but also differentially expressed in CRC, the intersection of 912 key module genes and 5,541 DEGs was calculated, resulting in the acquisition of 270 candidate genes (**Figure 1H**, **Supplementary Table 3**). The enrichment analysis of candidate genes revealed their involvement in crucial biological functions, highlighting their roles in complement and coagulation cascades, and the IL-17 signaling pathway (see **Additional File 1**: **Supplementary Figure S1A**). A total of 466 significant items were identified during the extension of the GO enrichment analysis, encompassing 401 biological processes (BPs) like response to molecules of bacterial origin, 22 cellular components (CCs) such as specific granules and tertiary granules, and 43 molecular functions (MFs) including cytokine activity and cytokine receptor binding (see **Additional File 1**: **Supplementary Figure S1B**). Collectively, our enrichment analysis deciphered the biological significance embedded in gene expression data, facilitating the understanding of the pathogenic mechanisms of CRC and its drug action pathways.

### Development of prognostic genes

**Figure 2:**
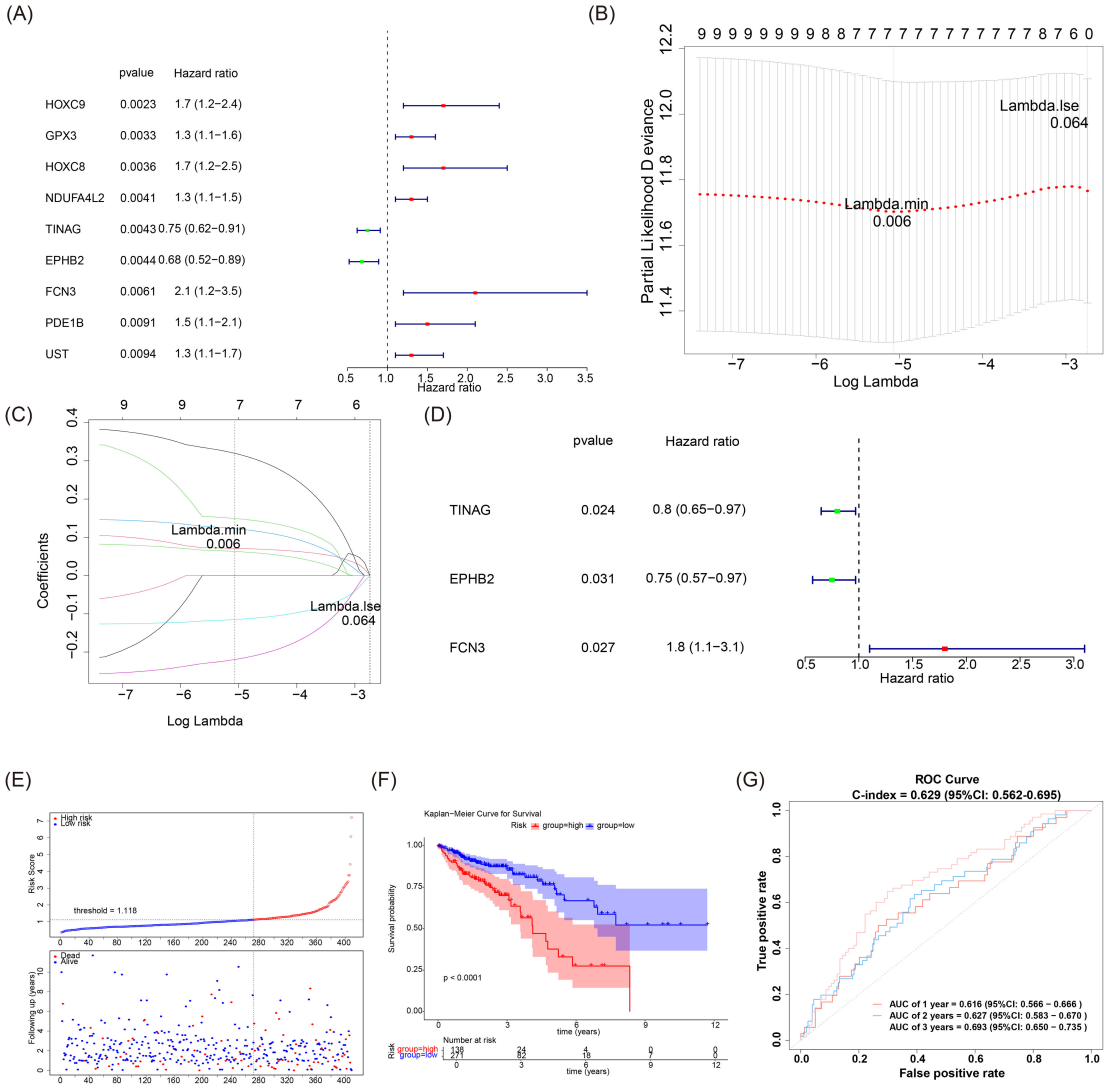
**(A)** Univariate Cox regression analysis of the forest plot. **(B, C)** Gene cross-validation error plot and coefficient plot derived from LASSO analysis. For both plots, the x-axis represents log(Lambda). The y-axes correspond to partial likelihood deviance and gene coefficients, respectively. **(D)** Multivariate Cox regression analysis of the forest plot. **(E)** Risk curve and scatter plot of the high-risk group in the training set. **(F)** KM survival curves of high (n = 138) and low-risk (n = 271) groups in the training set. The horizontal axis represents the follow-up time (in years), and the vertical axis represents the survival probability. The statistical significance test result of the survival difference between groups was p < 0.0001. **(G)** ROC curves for the training set at 1, 2, and 3 years.

By integrating univariate Cox analysis and machine learning algorithms, this study further derived prognostic genes from 270 candidate genes. The univariate analysis and PH test pinpointed nine genes associated with prognosis significantly (**Figure 2A**, **Supplementary Figure S2**). Using LASSO analysis, we constructed a model comprising seven genes in the training set (Log Lambdamin = 0.006), with further identification of three genes, TINAG, EPHB2, and FCN3, through multivariate Cox analysis (**Figures 2B–D**).

Subsequently, the risk scores of the samples were calculated according to Equation 1 the training set samples were segregated into two groups, based on the best threshold (bestthreshold=1.118) of risk scores, with a notably lower survival rate in the high-risk group (**Figure 2E**). Moreover, given the escalated risk score within the samples (**Figure 2F**), the risk graphs demonstrated a notable uptick in mortality. The ROC analysis of the risk model further affirmed its efficacy in forecasting the survival of CRC patients at 1, 2, and 3 years (**Figure 2G**). Bias correction of the risk scoring model was performed on the training set using bootstrap resampling (B = 1000). The results showed that the corrected AUC for 1–3 years was approximately 0.50, indicating a certain degree of uncertainty in the model’s prediction of short-term events. The C-index remained basically stable before and after correction (0.630 → 0.629), demonstrating that the model had good discriminative ability in ranking the overall survival time (**Supplementary Table 4**). In addition, through validation of the prognostic value, the construction of the risk model was reliable based on analyses on a testing set, consisting of an additional 175 individuals from the TCGA-CRC samples, and GSE87211 as an validation set (see **Additional File 1**: **Supplementary Figure S3**). Taken together, the reliability of predictions using this risk model underscored its overall quality and applicability, which was essential for assessing the effectiveness and feasibility of practical implementation. Collectively, all these results may provide valuable insights for clinical applications.

### Prognostic nomogram for CRC based on independent risk factors

**Figure 3:**
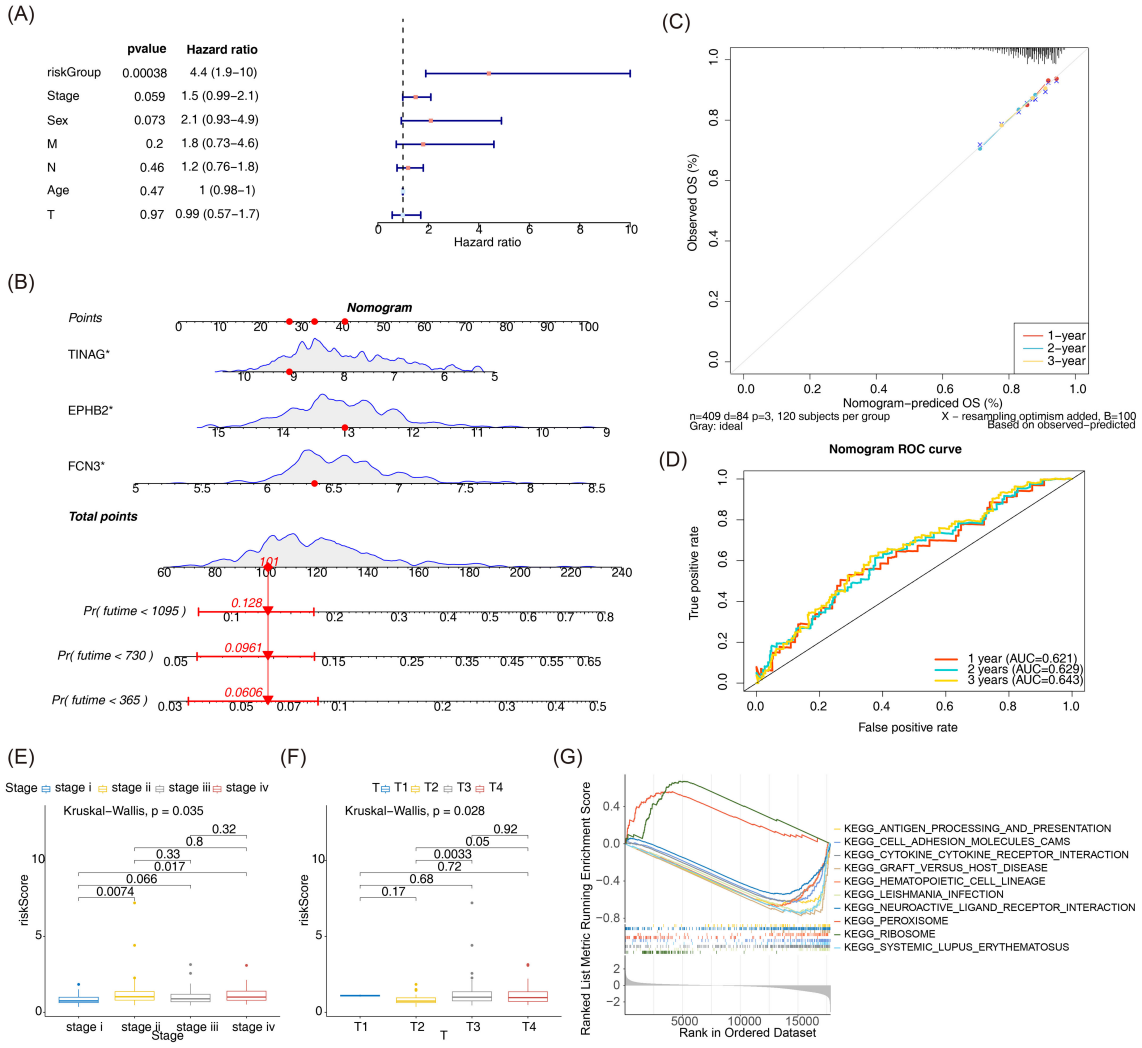
**(A)** Univariate Cox independent prognostic analysis forest plot. Risk score is an independent prognostic factor. **(B)** Nomogram constructed based on prognostic genes. The higher the total score in the figure, the lower the survival probability of the patient at 1, 2, and 3 years. **(C)** Calibration curve of the nomogram. The slopes of the calibration curves in 1, 2, and 3 years are all close to 1, indicating that the prediction accuracy of the nomogram is relatively high. **(D)** ROC curve of the nomogram. **(E, F)** Differential analysis of risk scores among different clinical subgroups. **(G)** Enrichment analysis of GSEA in the high-risk and low-risk groups.

Univariate Cox regression analysis was performed on clinical factors including the risk score, age, gender, tumor stage, and pathological TNM stage, and the results revealed that the risk score was an independent prognostic factor (**Figure 3A**). Then, a nomogram was developed by utilizing these independent prognostic factors, with a purpose of depicting the chance of survival for CRC patients at 1, 2, and 3 years (**Figure 3B**). Two algorithms were employed to further validate the effectiveness of the nomogram in forecasting CRC patients’ prognosis. The slope of the calibration curve, closely approaching 1, could suggest a high accuracy in predicting with the nomogram (**Figure 3C**). Additionally, the plotted ROC curve demonstrated that the AUC of the nomogram was above 0.6, indicating a favorable predictive effect of the nomogram model constructed in our study (**Figure 3D**).

According to further analysis of the distribution proportion of patients with each clinical characteristic in different groups, a greater part of patients in the high-risk group were aged over 60 and had M1, N1, T3, and stage II staging (see **Additional File 1**: **Supplementary Figure S4**). Additionally, significant discrepancies in risk scores were observed among the subgroups of stage and T staging (**Figures 3E, F**).

To explore the potential biological differences in patients between groups, GSEA was performed, with the results displayed in **Figure 3G**. Both groups presented remarkable enrichment in different biological functions and signaling pathways. The high-risk group showed upregulation of ribosomes and the peroxisome pathway, while downregulating pathways such as Leishmania infection, cytokine–cytokine receptor interaction, systemic lupus erythematosus, hematopoietic cell lines, cell adhesion molecule cams, and antigen processing and presentation. Through these analyses, we may get additional insights to comprehensively understand the underlying biological mechanisms, potentially enabling more effective stratification of patient risk.

### Prediction of risk models in immunotherapy

**Figure 4:**
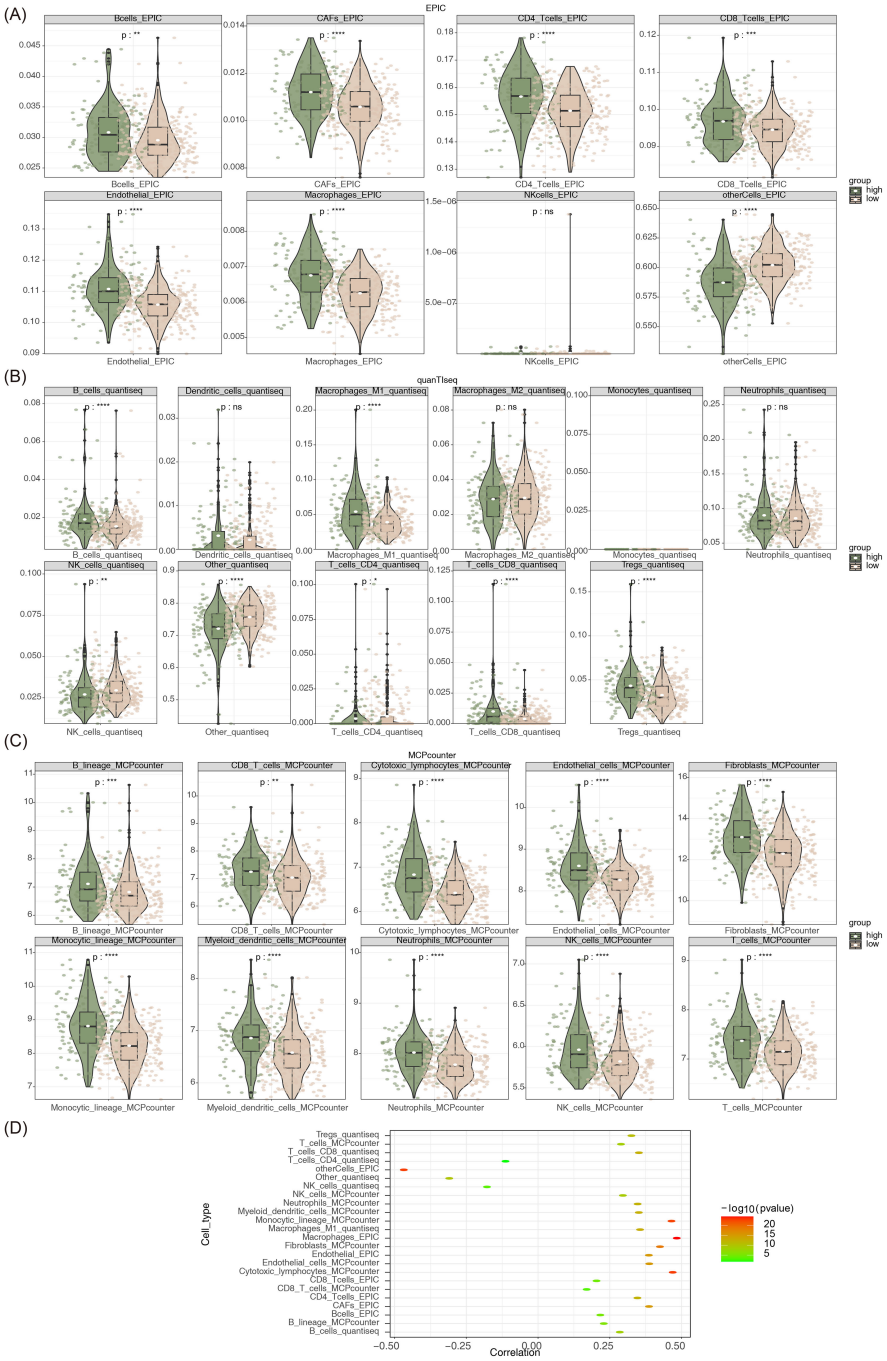
**(A-C)** Analysis of immune cell infiltration levels in the high- and low risk groups (EPIC, QUANTISEQ, and MCPCOUNTER). **(D)** Correlation analysis between risk score and differential immune cell infiltration. ns indicates *p*>0.05, * indicates *p* < 0.05, ** indicates *p* < 0.01, *** indicates *p* < 0.001, **** indicates *p* < 0.0001.

Utilizing the system’s own immune system to identify, target, and eradicate tumor cells, immunotherapy can stimulate, enhance, or reconstruct the patient’s innate immune response against cancer cells, representing an alternative therapeutic approach of great significance. Based on an advanced understanding on the tumor immune microenvironment, immunotherapy is assuming an increasingly crucial role in anticancer treatment (37). As shown by heatmaps of the results from the three immune infiltration algorithms (see **Additional File 1**: **Supplementary Figures S5A-C**), there were different levels of infiltration of multiple immune cells in the high- and low-risk groups. Moreover, using the MCPCOUNTER algorithm, all 10 immune cells showed notable disparities between groups, whereas only seven immune cells exhibited differences in the context of using QUANTISEQ and EPIC algorithms (**Figures 4A–C**). Results of the correlation analysis indicated that there were positive correlations of macrophages, cytotoxic lymphocytes, fibroblasts, and monocytic lineage with risk scores, whereas negative correlations in other cells (**Figure 4D**).

Furthermore, the track of tumor immune phenotypes is essential for comprehending cancer immune mechanisms and establishing biomarkers to predict responses to immunotherapy (38). Therefore, our study calculated the enrichment scores for each stage of the sequential events in the cancer-immunity cycle. Consequently, there were notable differences in gene enrichment scores for every step within both groups, and interestingly, the gene enrichment scores for each step were elevated in the high-risk group (see **Additional File 1**: **Supplementary Figure S6**). It implies significant regulatory influence of particular genes at each stage of cancer development and progression, which were pivotal in shaping the trajectory of the disease.

### Somatic mutations in CRC patients

**Figure 5:**
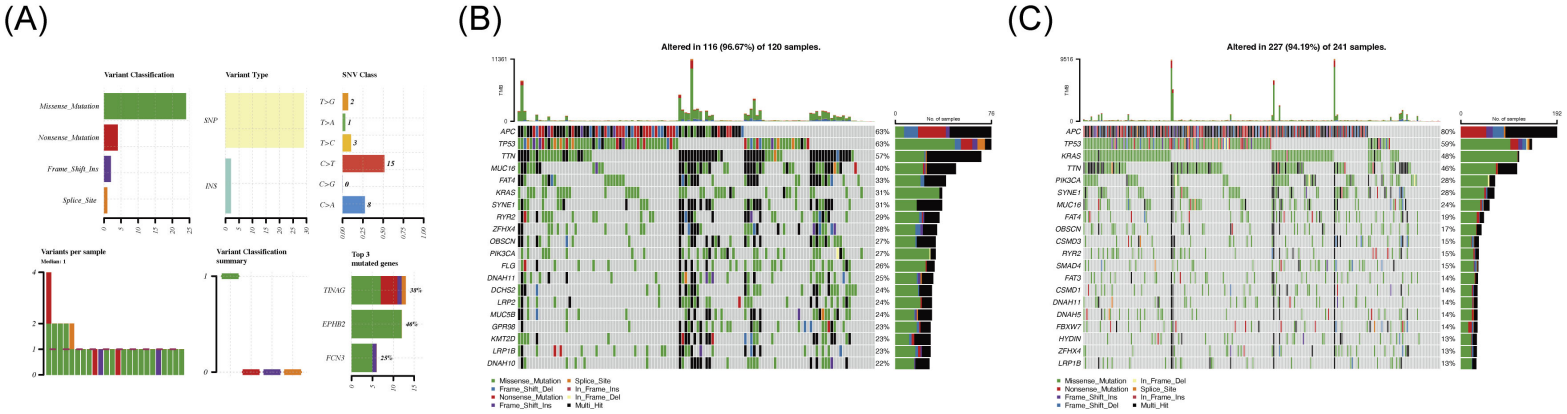
**(A)** Somatic mutation analysis. **(B)** Somatic mutations in high-risk group tumors. **(C)** Somatic mutations in low-risk group tumors.

Mutation analysis revealed that the majority of somatic mutations in patient samples from the training set were identified as missense mutations and single-nucleotide polymorphisms (SNPs), with C-to-T base substitutions exhibiting the highest frequency. The top three mutated genes were determined to be TINAG (38%), EPHB2 (46%), and FCN3 (25%) (**Figure 5A**). Subsequent tumor mutation profiling of the two risk groups showed that in the high-risk group, the top three most frequently mutated genes were APC (63%), TP53 (63%), and TTN (57%) (**Figure 5B**), whereas in the low-risk group, the leading mutations occurred in APC (80%), TP53 (59%), and KRAS (48%) (**Figure 5C**). Notably, missense mutations were identified as the predominant mutation type in both risk groups (**Figures 5B, C**). This mutation analysis has revealed the distribution patterns of missense mutations, C-to-T base substitutions, and key driver genes (such as APC, TP53, and KRAS) across different risk groups, providing molecular-level evidence for tumor prognosis stratification, personalized therapy, and biomarker development.

### Risk model correlated with the response of targeted therapy in CRC patients

**Figure 6:**
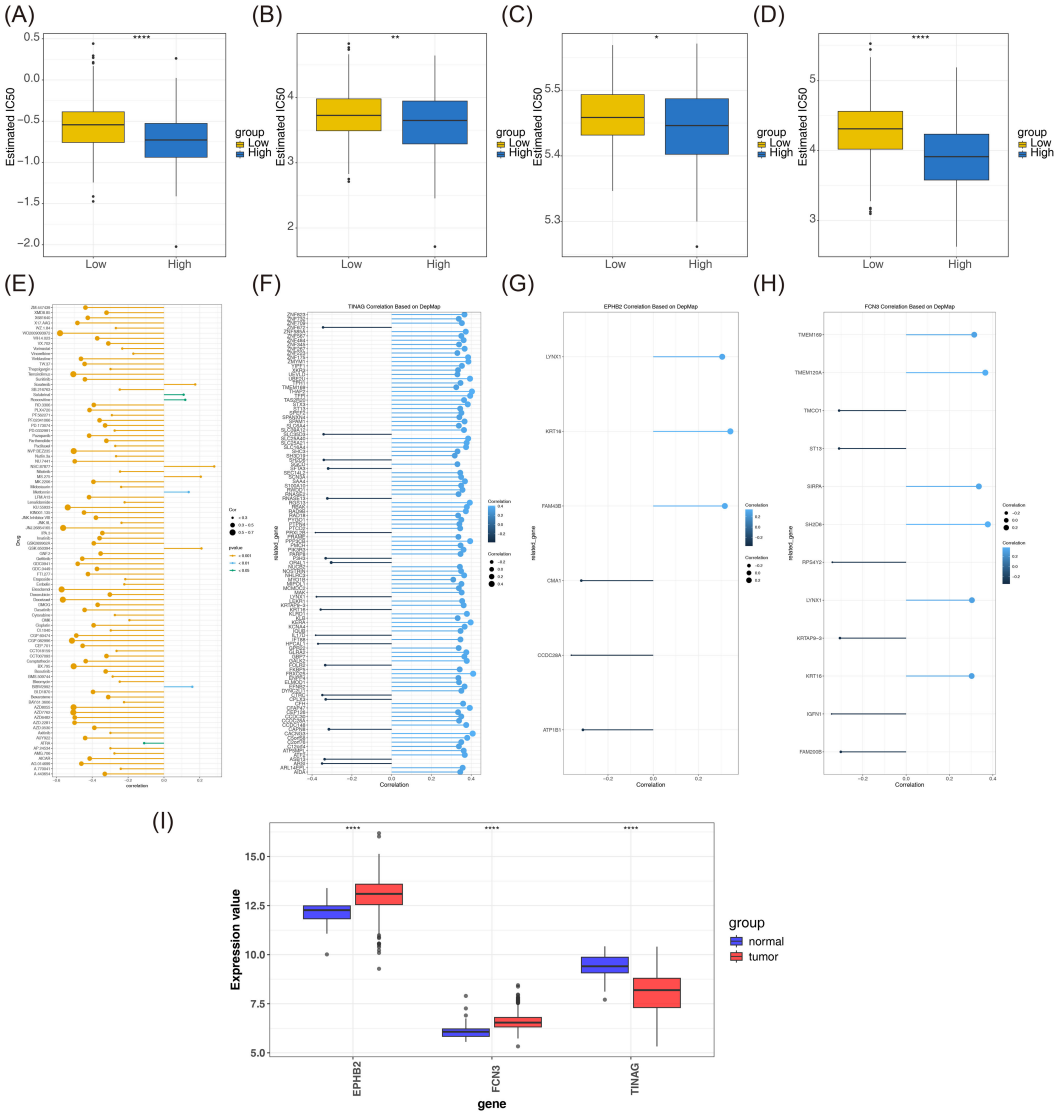
**(A-D)** Box diagram of drugs (A.443654, A.770041, ABT.888, and AG.014699) for the high- and low-risk groups. **(E)** Correlation analysis between drug sensitivity and risk score. **(F-H)** Related lollipop chart. **(I)** Analysis on the expression levels of prognostic genes. * represents *p* < 0.05, ** represents *p* < 0.01, and **** represents *p* < 0.0001.

Analysis on the differences in IC_50_ is a critical tool for determining drug sensitivity in personalized oncology. The IC_50_ value represents the drug concentration required to inhibit tumor cell growth, with lower values indicating higher sensitivity (39). An intergroup comparison of the IC_50_ values of 138 drugs revealed 92 drugs with statistically significant variances (*p* < 0.05). **Figures 6A–D** illustrates the relevancy between IC_50_ values and both groups for different drugs, including A.443654, A.770041, ABT.888, and AG.014699. Further correlation analyses on risk scores and drugs revealed that Sorafenib, Salubrinal, Roscovitine, NSC.87877, MS.275, Metformin, GSK.650394, and BIBW2992 exhibited direct correlations with risk scores. Conversely, WO2009093972 demonstrated an inverse correlation with risk scores, displaying the strongest correlation (**Figure 6E**).

Interestingly, the analysis of prognostic gene dependency showed that TINAG with 108 potential target genes had the strongest correlation with FBXO25, indicating a high level of dependency. EPHB2 had six potential target genes, exhibiting the strongest correlation with CCDC28A. FCN3 produced 12 potential target genes, and it was identified to have the strongest correlation with SH2D6 (**Figures 6F–H**). In the examination of the expression patterns of prognostic genes during disease progression, upregulation of EPHB2 and FCN3 as well as downregulation of TINAG were found in patients with CRC, demonstrating the trend of prognostic gene expression over the course of CRC (**Figure 6I**).

### High expression of prognostic gene EPHB2 verified by RT-qPCR and Western blotting

**Figure 7:**
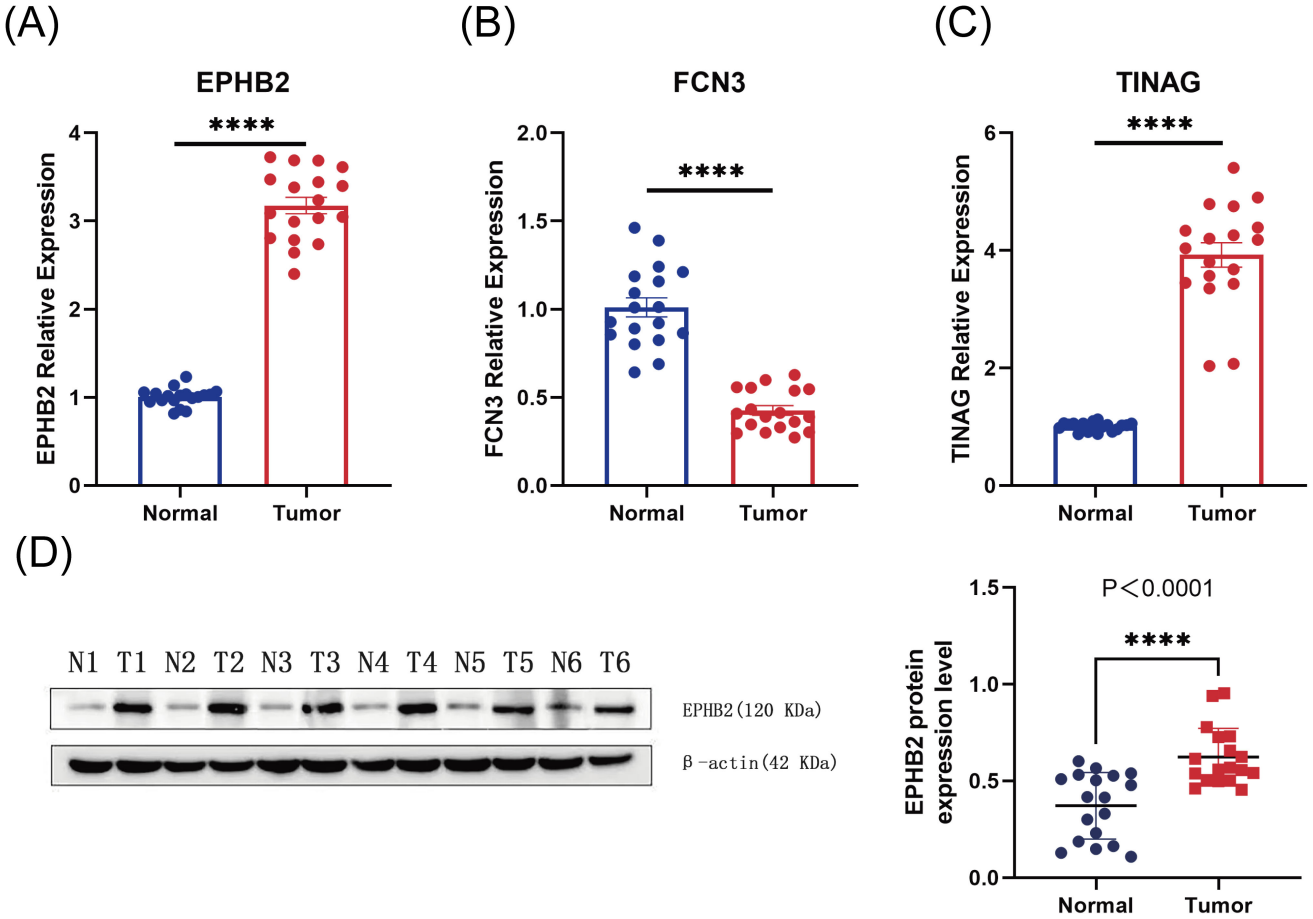
**(A-C)** Expression of prognostic genes in 18 sets of tissues. **(A)** EPHB2, **(B)** FCN3, and **(C)** TINAG. **(D)** Expression of EPHB2 in 18 pairs of tissues by Western blotting. **** represents *p* < 0.0001.

Firstly, we detected EPHB2, FCN3, and TINAG, three prognostic genes obtained from bioinformatics analysis, at the mRNA expression level, using the RT-qPCR method on 18 pairs of CRC and adjacent tissues collected clinically (**Table 2**). As depicted in **Figures 7A–C** regarding the differential expression levels of the three genes in clinical samples, the mRNA expression level of EPHB2 was significantly higher in CRC tissues than adjacent tissues (*P* < 0.05), whereas FCN3 and TINAG exhibited significantly reduced expression levels (both *P* < 0.05). Therefore, EPHB2, given its consistent expression level with that in the bioinformatics level, was selected for subsequent experiments. Further validation using Western blotting (**Figure 7D**) indicated significantly higher protein expression levels of EPHB2 in CRC tissues than in adjacent tissues (P<0.05). Once again, our study documented obviously higher expression levels of EPHB2, an MDM-RG, in CRC tissues than that in normal tissues.

**Table 2:** RT-qPCR sample information.

Number	Gender	Age	Location	TNM
1	Male	44	Ascending colon	T3N0M0
2	Male	53	Ascending colon	T2N0M0
3	Female	77	Ascending colon	T3N0M0
4	Male	74	Rectum	T3N0M0
5	Male	53	Rectum	T3N1M1
6	Female	59	Rectum	T3N0M0
7	Male	72	Rectum	T3N2M1
8	Female	41	Rectum	T3N2M0
9	Male	54	Rectum	T3N0M0
10	Male	74	Rectum	T4N2M0
11	Male	53	Rectum	T3N1M0
12	Male	65	Rectum	T4N1M0
13	Female	73	Ascending colon	T3N1M0
14	Male	58	Rectum	T3N1M1
15	Male	34	Descending colon	T4N2M1
16	Male	60	Ascending colon	T3N1M0
17	Female	61	Ascending colon	T3N1M0
18	Male	48	Rectum	T3N1M0

## Discussion

Given an annual increase in the incidence of CRC and unsatisfied treatment effect for advanced CRC, it highlights the necessity for exploring relevant prognostic genes of CRC (40). Mitochondrial DNA methylation has been documented to be critical in tumor occurrence, evolution, metastasis, and recurrence (41). Regarding tumor metabolism, mtDNA methylation can directly affect the efficiency of mitochondrial oxidative phosphorylation by modulating the expression of genes that encode key subunits of respiratory chain complexes. This change compels tumor cells to depend more heavily on glycolysis for their energy supply, a phenomenon known as the Warburg effect (21, 42, 43). This shift in metabolic state profoundly affects immune infiltration. On the one hand, metabolic waste products, such as lactic acid, which accumulate in the tumor microenvironment, can directly inhibit the antitumor function of cytotoxic T cells and NK cells (42, 44, 45). On the other hand, abnormal levels of mtDNA methylation may affect the stability of mitochondrial DNA, leading to its release into the cytoplasm. This can activate innate immune pathways, such as the cGAS-STING pathway, inducing the production of type I interferons. Consequently, this can lead to the recruitment and regulation of the infiltration and polarization state of immune cells, including dendritic cells and macrophages (46, 47). Therefore, mtDNA methylation acts as a crucial link that connects the intrinsic metabolism of tumor cells with the external immune microenvironment. In-depth research into this area will offer a new perspective for the development of innovative therapeutic strategies aimed at the tumor metabolism-immune axis. For the first time, this study screens for CRC related MDM-RGs, TINAG, EPHB2, and FCN3 by searching for TCGA-CRC transcriptome data. Then, the prognostic gene EPHB2 was further screened and validated using clinical tissues harvested by surgical resection.

To be specific, TINAG is a protein-encoding gene, also known as tubulointerstitial nephritis antigen, which is a renal tubular basement membrane protein (48). The expression of TINAG has been reported to be related to the development of kidney-related diseases, such as membranous nephropathy, interstitial nephritis, obstructive nephropathy (48), and renal clear cell carcinoma (49). Moreover, TINAG can promote cell proliferation of pancreatic cancer, serving as a prognostic indicator for this cancer (50). Given its nature, TINAG enables the construction of a basement membrane-related risk model that can effectively predict the prognosis of CRC patients (51).

In this study, FCN3 was identified as one of the MDM-RGs associated with CRC prognosis. Although FCN3 is best known for its role as an initiating molecule in the lectin pathway, regulating innate immunity and complement activation (52–54), its inclusion in this prognostic model suggests that it may indirectly influence mitochondrial function and epigenetic status by linking the immune microenvironment with cellular metabolism. Recent studies have demonstrated that the functions of the complement system and its components extend far beyond the traditional scope of immune defense. For instance, products of complement activation can directly or indirectly influence tumor cell survival signals and metabolic reprogramming (55, 56). Notably, FCN3-induced endoplasmic reticulum stress and ferroptosis are two cellular stress states closely linked to mitochondrial function (57). Endoplasmic reticulum stress can interact with mitochondria via the unfolded protein response, affecting mitochondrial membrane potential and reactive oxygen species production—factors reported to regulate mtDNA methylation (58). Therefore, we hypothesize that FCN3 may associate with mtDNA methylation through the following pathways: In CRC, alterations in FCN3 expression could modulate local immune responses or directly affect tumor cells, triggering cellular stress (e.g., endoplasmic reticulum stress) and subsequently disrupting mitochondrial homeostasis. This mitochondrial dysfunction may serve as an upstream signal, altering DNA methyltransferase activity or localization, ultimately influencing mtDNA methylation patterns and thereby contributing to tumor progression and patient prognosis. Although the direct molecular mechanism linking FCN3 to mtDNA methylation remains to be elucidated, our bioinformatics screening results and robust performance in prognostic models, combined with the aforementioned potential biological connections, strongly support further investigation of FCN3 as a valuable mtDNA methylation-associated indirect regulator.

Furthermore, EPHB2 is a transmembrane protein that mainly expresses pro-apoptotic transcription factors. It can mediate the regulation of the Wnt signaling and has a high interaction between EPHB2 receptor and its homologous specific EFNB1 ligand in gastric cancer, which is associated with poor prognosis (59–61). However, EphB2 expression has been reported to be dynamically regulated in different tumor progressions in multiple ways. Besides expression on tumor cells, EPHB2 can also be detected in monocytes, T cells, B cells, and some other immune cells (62). These characteristics partially determine the complexity of EPHB2 expression in tumors, resulting in potentially different roles in various tumors, and even within the same tumor due to various factors (62). For instance, EPHB2 is highly expressed in hepatocellular carcinoma, lung adenocarcinoma, gastric cancer, and CRC (59, 63–65) but lowly expressed in esophageal cancer and squamous cell carcinoma of the skin (66, 67). However, its expression remains controversial in gastric cancer and bladder cancer. In particular, EPHB2 is highly expressed in CRC, yet with an undetermined mechanism of action, which may be attributed to the intracellular environment, gene dependence and mutation, pathway mediation, and dual regulation of EPHB2 (62, 68).

In this study, TINAG and EPHB2, with HR below 1, were protective factors, whereas FCN3 was a promoting factor. EPHB2 was validated to be highly expressed in CRC, consistent with previous studies, highlighting its critical role in CRC. Our discovery of upregulated EPHB2 underscores its potential as a novel therapeutic target for CRC. Furthermore, further investigations should be conducted to clarify the role of this factor in CRC and its potential as a new therapeutic target for CRC.

Our study constructed a prognostic model integrating the three MDM-RGs (TINAG, EPHB2, and FCN3) associated with CRC prognosis, combined with further validation and evaluation of the effectiveness and accuracy of this prognostic model. Consequently, this prognostic model has significant application value in accurately predicting the prognosis of CRC patients.

With the creation of high- and low-risk groups, this study further conducted GSEA enrichment analysis, obtaining primary KEGG pathways related to each prognostic gene, such as ribosome, leishmania infection, cytokine and cytokine receptor interactions. To be specific, ribosomes are a collection of rRNA and ribosomal proteins that function in mRNA translation machines. Ribosomal biogenesis is a complex process controlled by multiple checkpoints and pathways, and abnormalities in ribosomal biogenesis may be observed owing to changes in these control points and processes (69). Generally, cancer cells contain special ribosomes that can promote oncogenic translation, contributing to an increased risk of malignant tumors (69). Ribosomal damage and its biological interference can both lead to the development of cancer, and interfering with protein synthesis can promote tumor cell death (70). Moreover, overactive ribosome biosynthesis can promote unrestricted cell proliferation (71). Similarly, as suggested previously, ribosomal stress and functional changes may participate in the development of thyroid cancer, leukemia, and also CRC (72, 73). Therefore, MDM-RGs screened in our study may regulate various biological processes to affect the growth of CRC.

To acquire a quantitative method for predicting the contribution of different prognostic genes to CRC, this study further constructed a nomogram, with risk score as an independent prognostic factor. Considering the role in innate immunity by activating the lectin complement pathway, FCN3 showed a greater contribution to prediction within the constructed nomogram. Our study also compared clinical features and found significant differences in risk scores between stage and T stage subgroups. According to the corresponding results, in the early stages of CRC (e.g., stages I and II) with the presence of local lesions usually, lower risk scores might reflect less tumor spread and better prognosis; in contrast, late stage (e.g., stage III and IV) often has local lymph node metastasis or distant metastasis, with the observation of higher risk scores in the involved patients, suggesting a higher degree of disease severity and poor prognosis. In terms of the impact of T staging, the tumor is still confined to the intestinal wall at T1 and T2 stages, usually accompanied by a lower risk score. T3 and T4 stages may be detected with tumor penetration of the intestinal wall and potential invasion of adjacent organs or structures, resulting in higher risk scores, requiring in more invasive treatment possibly.

Our study also uncovered a significant correlation between immune infiltration in CRC—particularly macrophages, cytotoxic lymphocytes, fibroblasts, monocytic lineage cells, and other cells, with risk scores. Macrophages can be involved in maintaining tissue health and repair processes, besides acting as key regulators of immune response (74). Cytotoxic lymphocytes are an important class of cells in the immune system that directly recognize and kill infected cells, diseased cells, or tumor cells, thereby preventing the invasion of various pathogenic microorganisms and abnormal cells (75–78). Both types of cells were positively correlated with risk scores in our study. In addition, other cells represent a population of cells other than the known major cell types in the immune system (e.g., T cells, B cells, and macrophages) and were negatively correlated with risk scores. NK cells are the main infiltrating cells of CRC that may restrict the invasion of cancer cells into tissues (79), showing higher infiltration in our high-risk group, thus potentially regulating CRC occurrence. Mechanistically, allogeneic NK cells can be engaged in favorable myeloid cross talk, display effective antitumor activity, and, when combined with R848, may induce a pro-inflammatory shift of the microenvironment of CRC (80). Therefore, our thorough investigation on clarifying differences in immune cells between high- and low-risk groups may provide novel insights into disease pathogenesis, prognosis, therapeutic response, and even immune regulation processes, thereby boosting the improvement of disease diagnosis and treatment.

Our subsequent comparison emphasized the differences in IC_50_ values of common chemotherapeutic agents between high- and low-risk groups. As a result, there were significant differences in IC_50_ values of 92 drugs between groups. The IC_50_ of Veliparib (ABT-888), a PARP inhibitor, was lower in the high-risk group, which can be used to treat and may be more effective for managing CRC (81). The BCL-XL targeting agent, ABT-263, can increase intracellular ROS levels and can augment the therapeutic efficacy of this BH3 mimetic by targeting antioxidant pathways (82). Collectively, the lower IC_50_ values of ABT-888 and ABT-263 in the high-risk group support their stronger efficacy in treating high-risk patients.

In addition, our experiments also screened for the dependence of prognostic genes in CRC cells and identified prognostic genes that were highly dependent on CRC cell lines, which could serve as potential therapeutic targets for CRC. TINAG, EPHB2, and FCN3 were identified as critical signaling molecules and potential targets for the treatment of CRC, which deserve further exploration to confirm their efficacy and mechanisms more comprehensively, and in particular, to evaluate their effectiveness and safety as targeted therapy strategies in clinical trials. In the future, it is possible to achieve higher precision and personalized levels of treatment for CRC by targeting these genes and integrating them with modern cancer treatment approaches. Simultaneously, it is expected that with the continuous deepening of research, these genes will provide more therapeutic options for CRC patients, improving therapeutic outcomes and bringing more positive prognoses to patients.

This study, based on bioinformatics analysis, first screened and validated the prognostic value of three MDM RGs, TINAG, EPHB2, and FCN3, in CRC. It should be pointed out that the AUC value of the three-gene risk model we constructed in the training set, testing set, and external validation set is approximately 0.6, and its individual predictive discrimination ability may not be sufficient as an independent clinical diagnostic tool. However, the core value of this study lies in its exploratory nature—we aim to extract a concise and novel gene tag related to a specific molecular mechanism (mtDNA methylation) from complex tumor biology. Despite limited prediction accuracy, the model successfully stratified patients into risk subgroups with significant differences in survival, immune infiltration characteristics, and drug sensitivity. The consistency observed across multiple datasets strongly suggests that the biological pathways represented by these three genes play an important role in the evolution of CRC. Therefore, the current significance of this model is more focused on providing a new perspective for the molecular subtyping of CRC, and its future clinical application potential may lie in combining with traditional indicators such as TNM staging to jointly optimize patient risk assessment and management strategies.

This study has some limitations that need to be addressed. Firstly, the direct correlation between the three prognostic genes and mitochondrial DNA methylation necessitates further experimental investigation. Secondly, the sample size for clinical validation is relatively small, and experiments to protein levels of FCN3 and TINAG have not yet been assessed. Additionally, the predictive performance and practical application value of the risk model and nomogram we constructed require enhancement for use in clinical settings. Consequently, we intend to further explore the direct regulatory relationship between TINAG, EPHB2, FCN3, and mitochondrial DNA methylation through subcellular localization, mtDNA methylation site detection, and gene knockdown/overexpression experiments in the future. Furthermore, we plan to expand the clinical sample cohort, conduct multicenter validation, and systematically verify the protein expression levels of FCN3 and TINAG using Western blotting and immunohistochemical techniques. Finally, we aim to incorporate more clinical variables or novel molecular markers, optimize model construction using more sophisticated machine learning algorithms, and carry out prospective clinical studies to effectively assess its predictive accuracy and clinical application potential, thereby facilitating its transition into a clinical tool.

To sum up, this study screens for MDM-RGs in CRC based on bioinformatics analysis and explores potential therapeutic targets. TINAG, EPHB2, and FCN3 are identified as three representative MDM-RGs that exhibit significant correlations with various immune cell infiltrations, with further validation at the mRNA and protein levels. These three screened MDM-RGs are expected to become new targets for CRC prognostic evaluation and treatment. Our future research will continue to focus on the clinical roles of TINAG, EPHB2, and FCN3 through higher-quality research.

## Data Availability

The original contributions presented in the study are included in the article/**Supplementary Material**. Further inquiries can be directed to the corresponding author.

## Supplementary material

Supplementary Figure 1 **(A)** Tree diagram of KEGG enrichment. The Kyoto Encyclopedia of Genes and Genomes (KEGG) pathway map was obtained from KEGG(https://www.kegg.jp). KEGG is a publicly available resource under the terms of the academic use license (20-22) **(B)** Tree diagram of GO enrichment.

Supplementary Figure 2 PH test result chart.

Supplementary Figure 3 **(A)** Risk curve and scatter plot of the high-risk group in the testing set. **(B)** KM survival curves of high (n = 98) and low-risk (n = 77) groups in the testing set. **(C)** ROC curves for the testing set at 1, 2, and 3 years. **(D)** Risk curve and scatter plot of the high-risk group in the external validation sets. **(E)** KM survival curves of high (n = 82) and low-risk (n = 108) groups in the external validation sets. **(F)** ROC curves for the external validation sets at 1, 2, and 3 years.

Supplementary Figure 4 **(A-F)** Analysis of the Distribution Ratio of Clinical Characteristics in High and Low Risk Groups.

Supplementary Figure 5 **(A-C)** Analysis of immune cell infiltration levels in high and low risk groups (EPIC, QUANTISEQ, and MCPCOUNTER).

Supplementary Figure 6 **(A-G)** Anti-tumor immune analysis of high-risk and low-risk groups.

Supplementary Table 1 Details of MDM-RGs.

Supplementary Table 2 List of 912 key module genes.

Supplementary Table 3 List of 270 candidate genes.

Supplementary Table 4 The risk model was verified in the training set through the bootstrap method.
