# Supplementary figures and images for: Novel mtDNA methylation-associated prognostic signatures in colorectal cancer
Source: Front Oncol. 2026 Jan 2;15:1684770. doi: 10.3389/fonc.2025.1684770 (PMC12807941; doi:10.3389/fonc.2025.1684770)

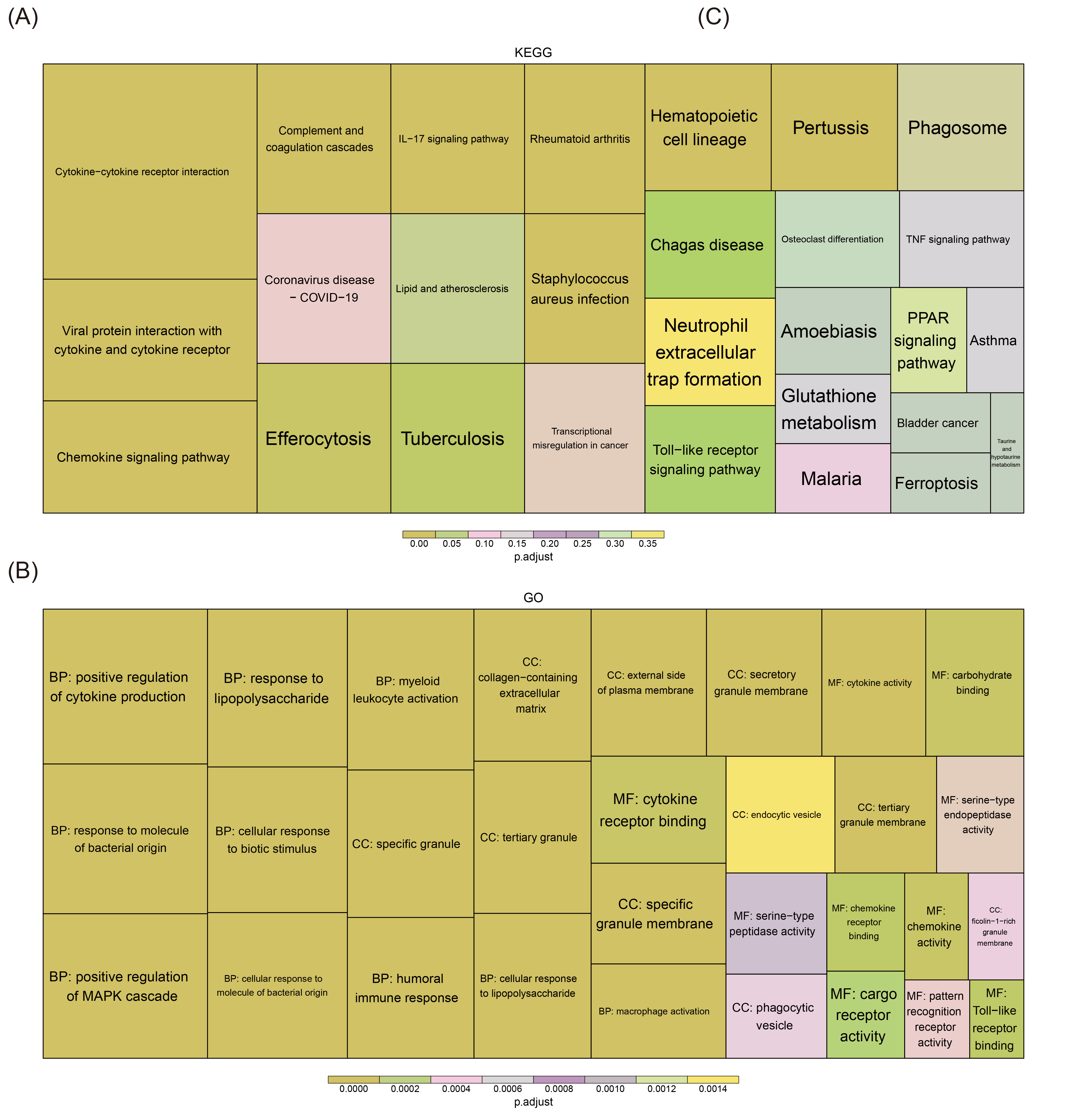

Supplement: Supplementary Figure 1 — (A) Tree diagram of KEGG enrichment. The Kyoto Encyclopedia of Genes and Genomes (KEGG) pathway map was obtained from KEGG(https://www.kegg.jp). KEGG is a publicly available resource under the terms of the academic use license (20-22) (B) Tree diagram of GO enrichment. [file Image1.tif]

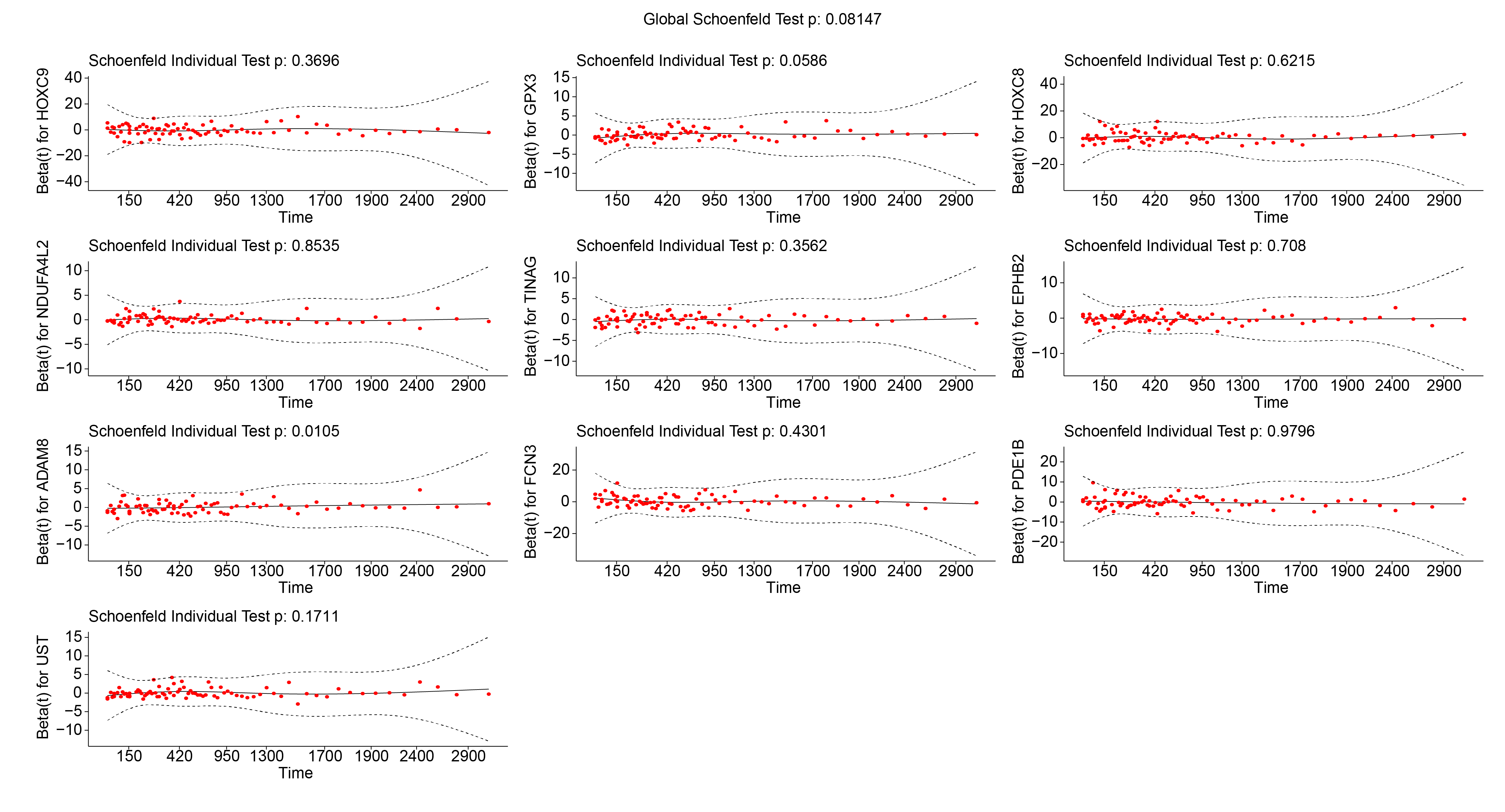

Supplement: Supplementary Figure 2 — PH test result chart. [file Image2.tif]

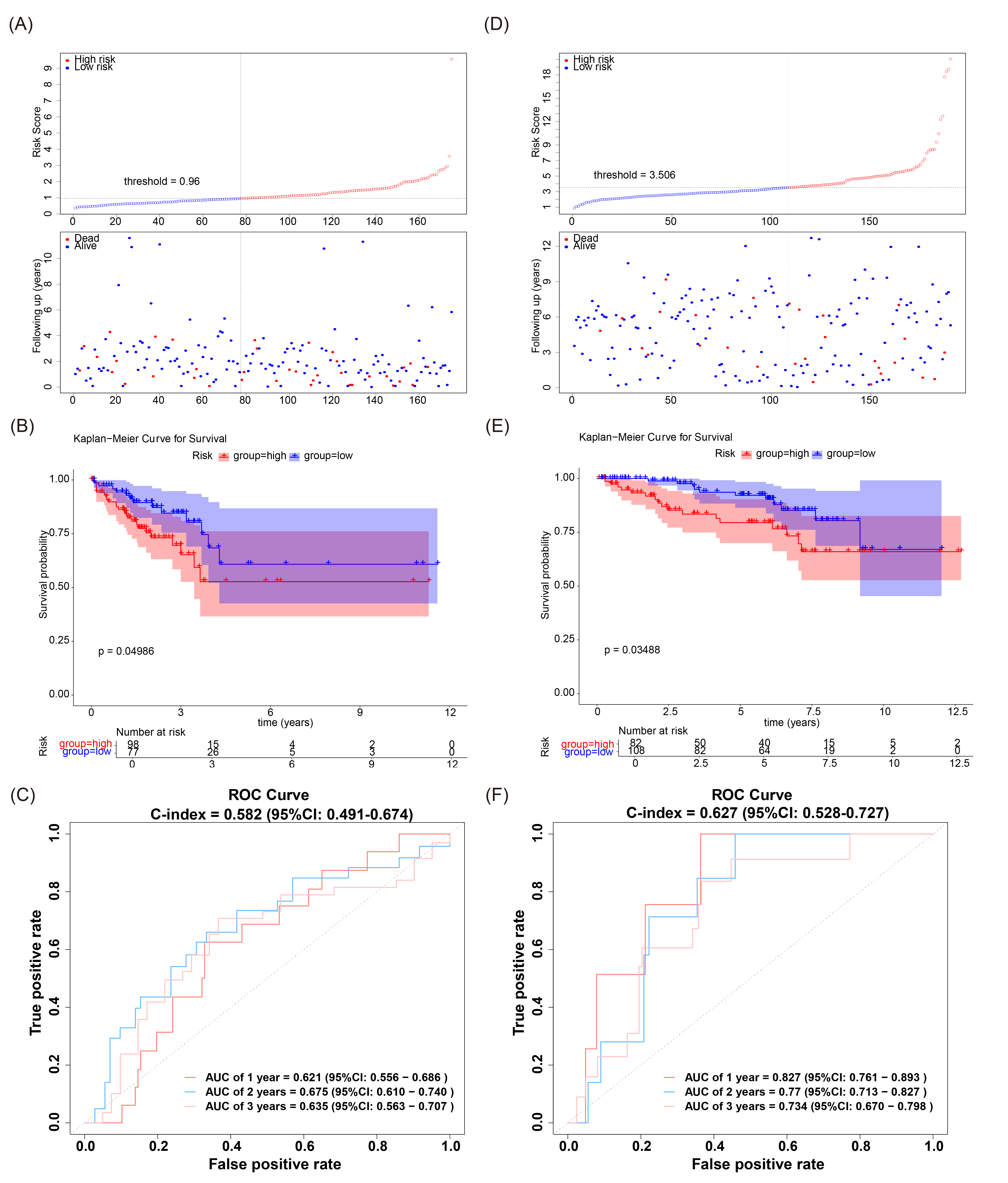

Supplement: Supplementary Figure 3 — (A) Risk curve and scatter plot of the high-risk group in the testing set. (B) KM survival curves of high (n = 98) and low-risk (n = 77) groups in the testing set. (C) ROC curves for the testing set at 1, 2, and 3 years. (D) Risk curve and scatter plot of the high-risk group in the external validation sets. (E) KM survival curves of high (n = 82) and low-risk (n = 108) groups in the external validation sets. (F) ROC curves for the external validation sets at 1, 2, and 3 years. [file Image3.tif]

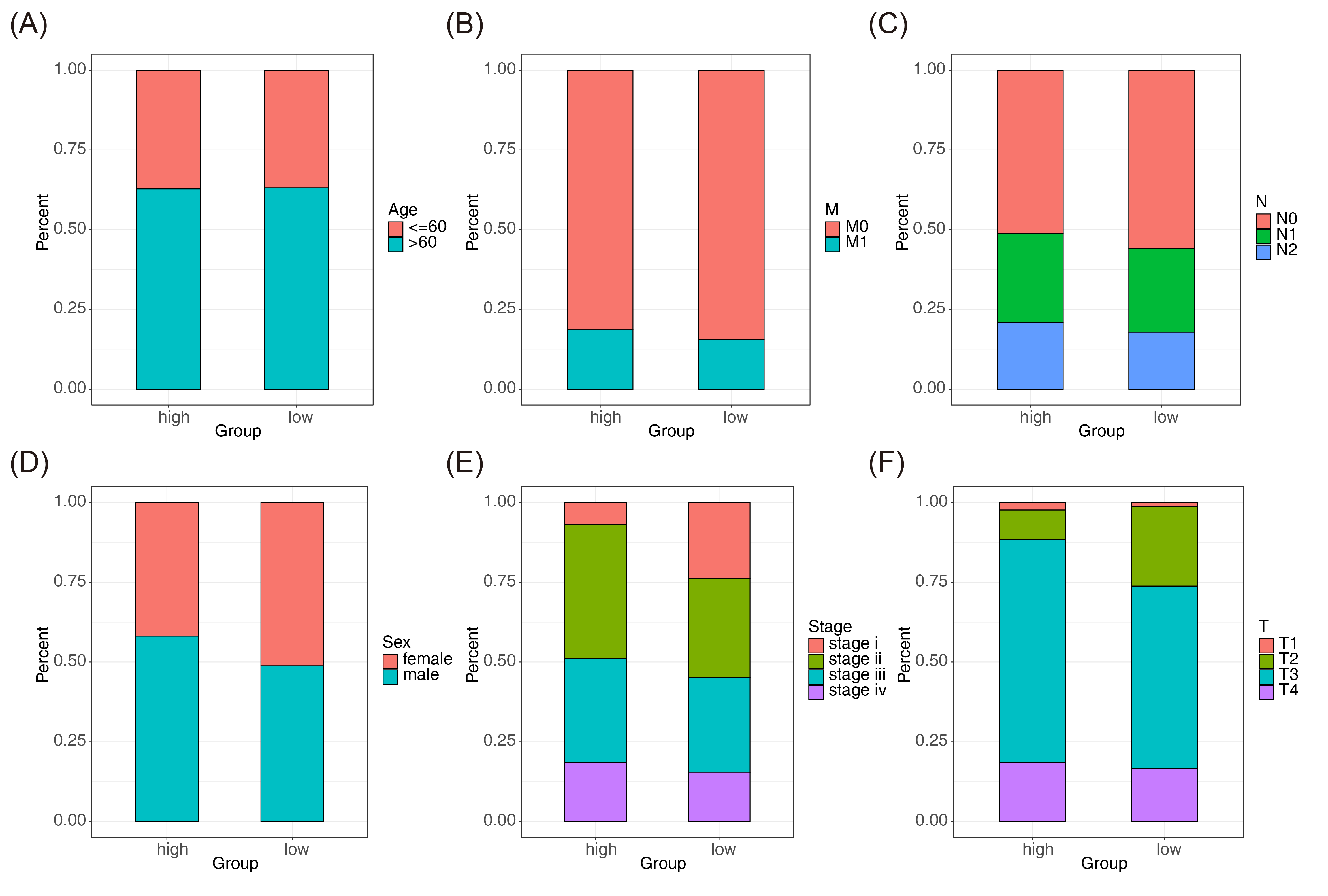

Supplement: Supplementary Figure 4 — (A-F) Analysis of the Distribution Ratio of Clinical Characteristics in High and Low Risk Groups. [file Image4.tif]

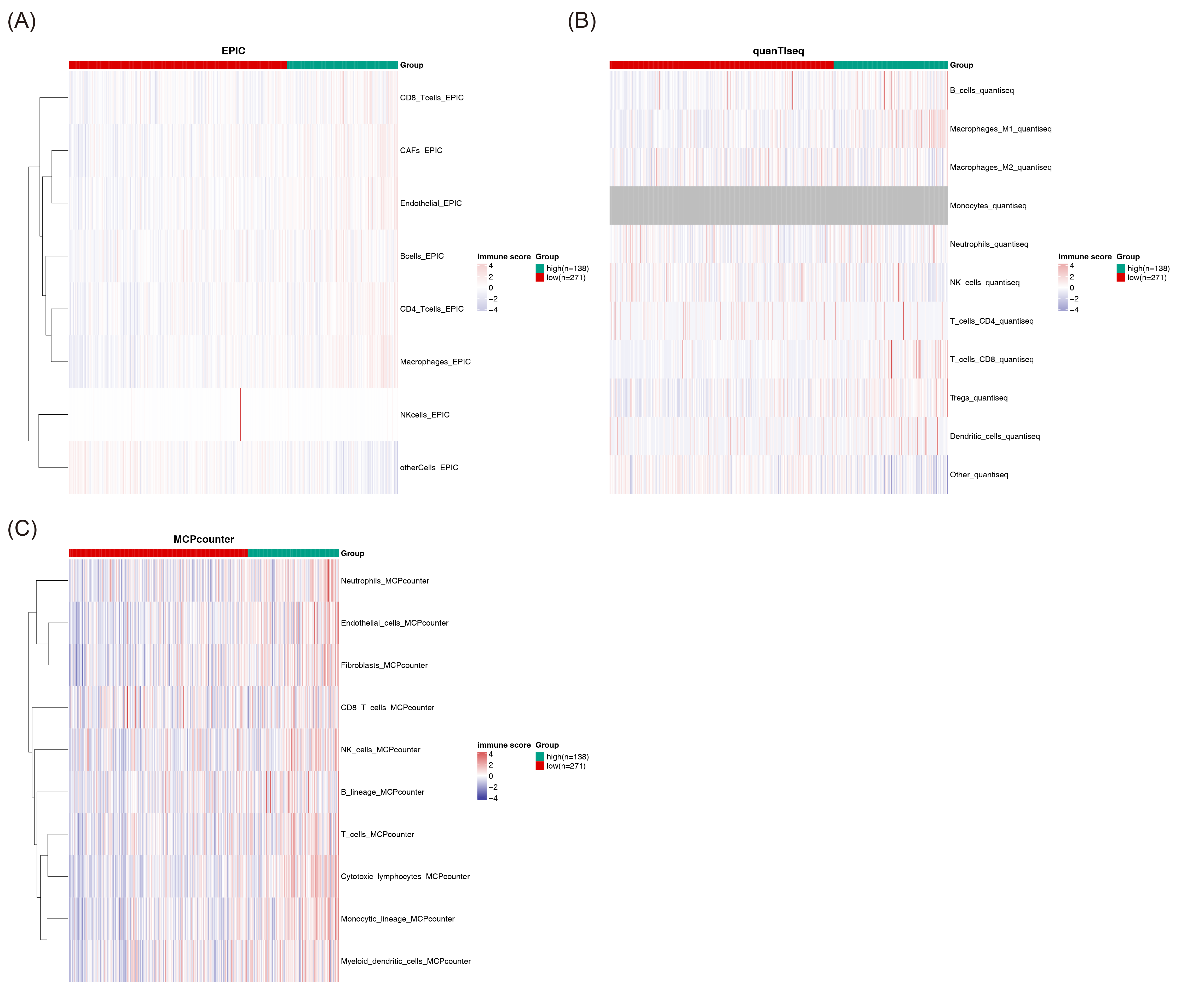

Supplement: Supplementary Figure 5 — (A-C) Analysis of immune cell infiltration levels in high and low risk groups (EPIC, QUANTISEQ, and MCPCOUNTER). [file Image5.tif]

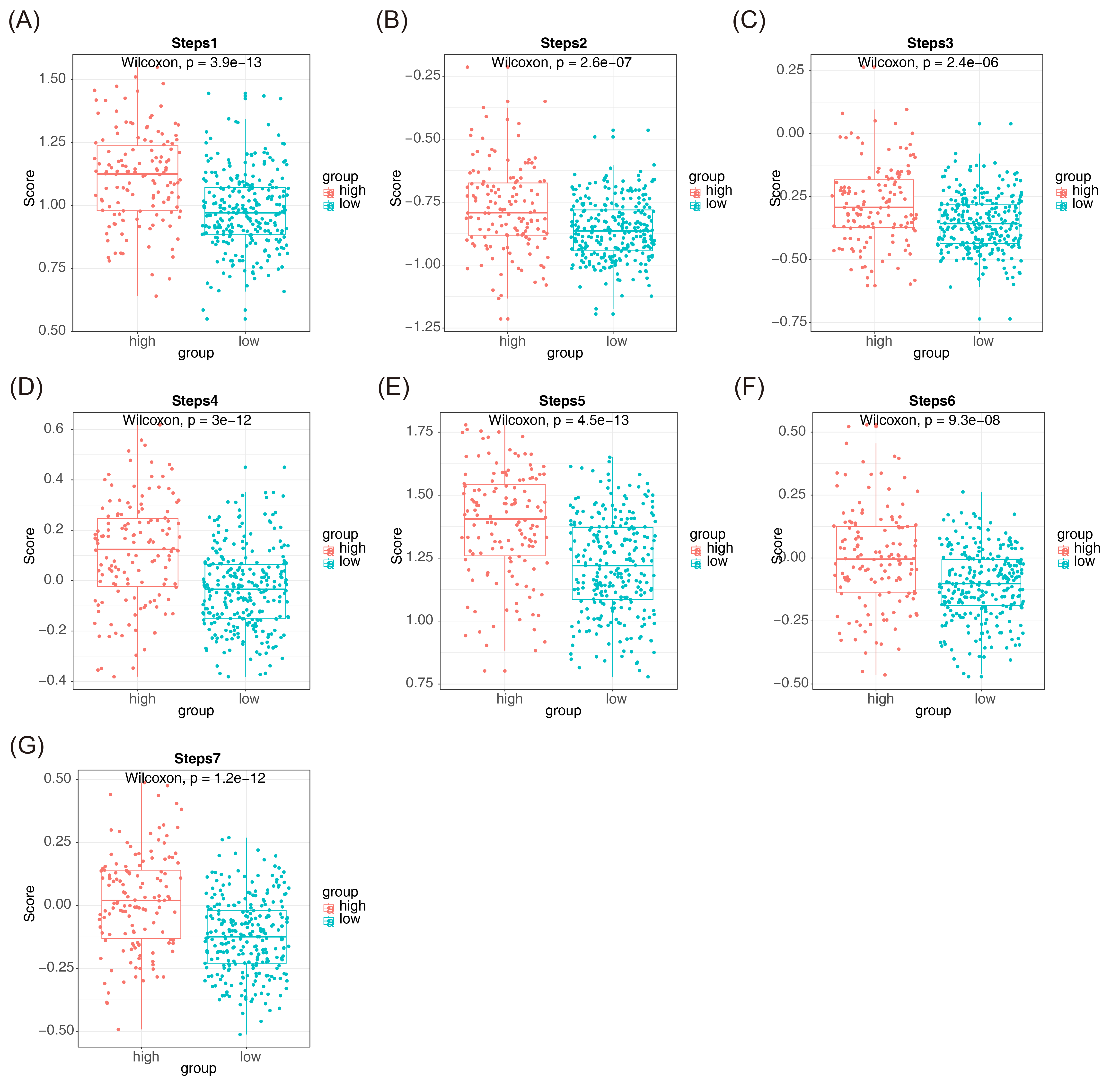

Supplement: Supplementary Figure 6 — (A-G) Anti-tumor immune analysis of high-risk and low-risk groups. [file Image6.tif]
